# Concentric chiral nematic polymeric fibers from cellulose nanocrystals†

**DOI:** 10.1039/d1na00425e

**Published:** 2021-08-11

**Authors:** Arash Momeni, Christopher M. Walters, Yi-Tao Xu, Wadood Y. Hamad, Mark J. MacLachlan

**Affiliations:** Department of Chemistry, University of British Columbia 2036 Main Mall Vancouver British Columbia V6T 1Z1 Canada mmaclach@chem.ubc.ca; Transformation and Interfaces Group, Bioproducts Innovation Centre of Excellence, FPInnovations 2665 East Mall Vancouver British Columbia V6T 1Z4 Canada; Stewart Blusson Quantum Matter Institute 2355 East Mall Vancouver British Columbia V6T 1Z4 Canada; WPI Nano Life Science Institute, Kanazawa University Kanazawa 920-1192 Japan; UBC BioProducts Institute 2385 East Mall Vancouver British Columbia V6T 1Z4 Canada

## Abstract

Hierarchical biological materials, such as osteons and plant cell walls, are complex structures that are difficult to mimic. Here, we combine liquid crystal systems and polymerization techniques within confined systems to develop complex structures. A single-domain concentric chiral nematic polymeric fiber was obtained by confining cellulose nanocrystals (CNCs) and hydroxyethyl acrylate inside a capillary tube followed by UV-initiated polymerization. The concentric chiral nematic structure continues uniformly throughout the length of the fiber. The pitch of the chiral nematic structure could be controlled by changing the CNC concentration. We tracked the formation of the concentric structure over time and under different conditions with variation of the tube orientation, CNC concentration, CNC type, and capillary tube size. We show that the inner radius of the capillary tube is important and a single-domain structure was only obtained inside small-diameter tubes. At low CNC concentration, the concentric chiral nematic structure did not completely cover the cross-section of the fiber. The highly ordered structure was studied using imaging techniques and X-ray diffraction, and the mechanical properties and structure of the chiral nematic fiber were compared to a pseudo-nematic fiber. CNC polymeric fibers could become a platform for many applications from photonics to complex hierarchical materials.

## Abstract 2

A cellulose nanocrystal liquid crystalline suspension was mixed with monomers and confined to a capillary tube. After photopolymerization, a fiber with a single-domain concentric chiral nematic structure throughout the length of the fiber was obtained.

## Graphical abstract

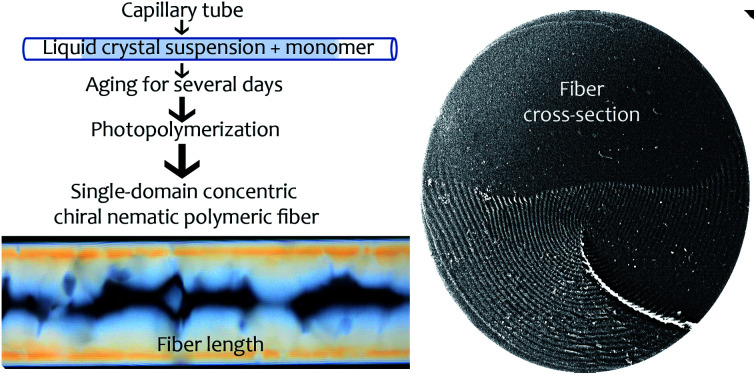

## Introduction

Biological materials, such as osteons and plant cell walls, have complicated hierarchical structures, with organization over many length scales.^1–3^ Each level in the hierarchy represents an increase in organizational complexity. At the first length scale, molecular- and nano-sized units co-assemble to form highly ordered micron-sized units. These micron-sized units interact in sophisticated ways to form larger structural units. This hierarchical fabrication process continues to the macroscopic scale, resulting in a material with unique properties. Mimicking the bottom-up construction of hierarchical materials at the nano/micron scale has remained elusive, but we show here that combining liquid crystals and polymerization techniques within confined spaces could be a strategy to recreate such complex structures in artificial systems.

**Fig. 1:**
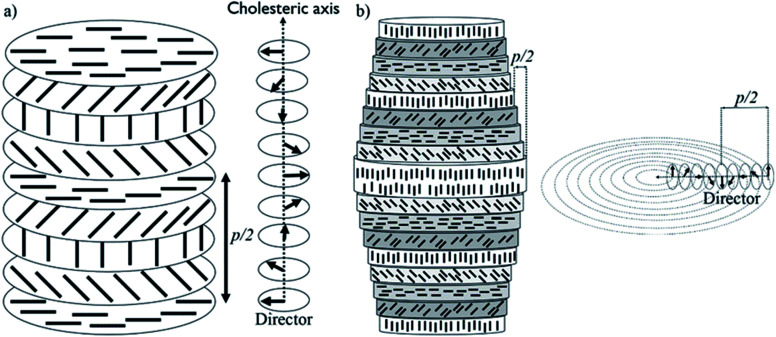
(a) Planar chiral nematic structure of CNCs and its director field; (b) concentric cylindrical chiral nematic structure of CNCs and its director field. The black rods indicates individual CNCs.

Cellulose nanocrystals (CNCs) are nano-sized spindle-shaped crystalline cellulose particles obtained by treatment of cellulosic biomass with strong mineral acids. When they are prepared with sulfuric acid, the CNC particles are covered with sulfate half-ester groups that give them a surface charge and lead to colloidal stability in water.^4^ Importantly, CNCs spontaneously form a chiral nematic (also called cholesteric) lyotropic liquid crystal above a critical concentration in water. Initially, liquid crystalline anisotropic droplets,^5^ called tactoids, spontaneously nucleate at CNC concentrations above ∼3 wt%.^6^ CNC tactoids are the intermediate state bridging the isotropic phase and the macroscopic liquid crystalline phase with longer range order.^7^ Upon further concentration, the CNCs form a continuous chiral nematic phase. In this chiral nematic structure, CNC spindles are aligned such that the liquid crystal director rotates through the liquid crystal along the cholesteric axis, as shown in Fig. 1a. The cholesteric pitch (*p*) is the distance needed for the director to rotate 360°.

When the lyotropic liquid crystal suspension of CNCs is dried into a film, the chiral nematic organization of the CNCs can be preserved in the solid state. If the pitch matches the wavelength of visible light, then the films can appear iridescent. Interesting materials have been developed based on the liquid crystalline nature of CNCs, including films,^8,9^ microcapsules,^10^ 3D printer inks,^11^ hydrogels,^12,13^ aerogels,^14^ and hybrid composites.^15,16^ The chiral nematic structure of CNCs has also been used as a template to form other chiral nematic materials such as chiral nematic silicate glasses.^17^

CNC structures developed by evaporation-induced self-assembly lack long-range order, do not have a uniform pitch value, and are multi-domain with cholesteric axes pointing in different directions in different regions.^18,19^ This is largely due to evaporation-induced flow, non-uniform evaporation, and uncontrolled liquid crystalline nucleation sites and growth. Magnetic fields^20,21^ and slow evaporation^22^ have been applied to improve the long-range order in CNC-based structures, but both techniques are slow and only simple planar structures have been reported.^20–22^ The organization of CNC particles can be manipulated by confining CNC suspensions in small spaces. For example, uniformly aligned photonic films were reported for CNC suspensions dried in a thin rectangular capillary.^23^ Furthermore, concentric spherical multi-shell structures with radially oriented cholesteric axes were reported for CNCs confined to microdroplets.^24,25^ In this paper, we show that by confining CNC aqueous solutions inside a cylindrical capillary tube, a long-range-ordered single domain of CNCs with chiral nematic structure can be obtained. The ordered structure is then locked in place by a polymerization technique, resulting in fibers with a concentric chiral nematic structure (Fig. 1b). We studied the effect of tube orientation, CNC concentration, CNC type, salt addition, capillary tube size, and time on the organization of the material. Polarized optical microscopy (POM), electron microscopy, confocal microscopy, 2D X-ray diffraction (2D-XRD), and tensile test measurements were applied to demonstrate the ordered structure and its properties. We discuss how a simple confinement approach could be applied to obtain well-ordered hierarchical structures with a wide range of potential applications.

## Results and discussion

**Fig. 2:**
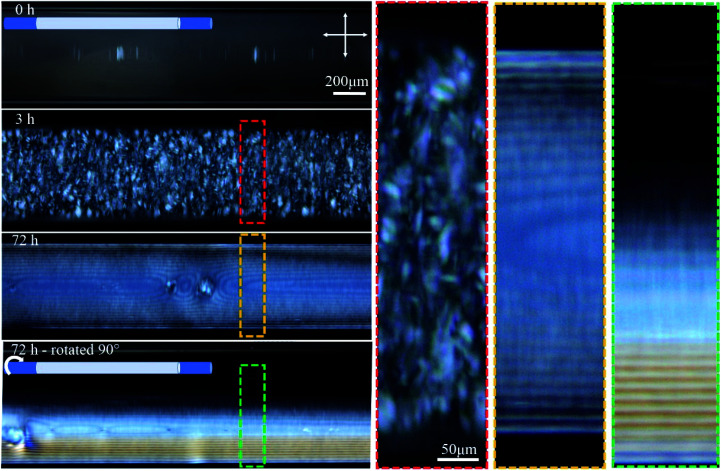
Time-series POM images of the 0.4 mm capillary tube filled with 4.5 wt% CNCs, and aged horizontally as shown in the cartoon. Colored dashed boxes show high magnification images. Crossed arrows show the polarizer/analyzer configuration. At 72 h, the tube was also imaged after rotating 90° around its long axis as schematically shown. The apparent inner diameter of the tubes in these images is 0.55 mm while the actual inner diameter is 0.4 mm. This discrepancy originates from light refraction through the cylindrical object (see Fig. S1, ESI†).

This study stemmed from the observation that filling a capillary tube (0.4 mm inner diameter) with 4.5 wt% CNC solution resulted in the formation of birefringent periodic lines parallel to the length of the tube when observed between crossed polarizers using POM (Fig. 2). The lines are evenly spaced and resemble the characteristic fingerprint lines observed in chiral nematic CNC structures using POM, where the distance between the lines is equal to one-half pitch (*p*/2).^26^

Temporal POM images of the capillary tube filled with 4.5 wt% CNC suspension show that it takes time for the ordered structure to form (Fig. 2). Immediately after filling the capillary, the solution was completely isotropic and the sample appeared dark when viewed between crossed polarizers. After 3 h, tactoids became visible, but disappeared over 72 h as they coalesced into a continuous liquid crystalline phase, which was evident in POM images as periodic dark lines that continue throughout the length of the tube. The same part of the tube was then imaged after rotating the tube 90° around its long axis, as shown in Fig. 2. Interestingly, the top half of the tube was dark (isotropic) and the bottom half showed the straight parallel lines. This observation suggests that tactoids settled to the bottom of the tube due to gravity before coalescence, and the periodic structure is only occupying the bottom half of the tube interior. Tactoid sedimentation is also observed in vertical orientation of the tube as will be discussed in the next paragraph. In Fig. 2, the distance between periodic lines in both views are equal to 14 ± 2 μm. Color that is apparent in the rotated view is due to the increase of the thickness as more CNCs are in the light path, as described by the Michel-Levy chart for birefringence.^27^

**Fig. 3:**
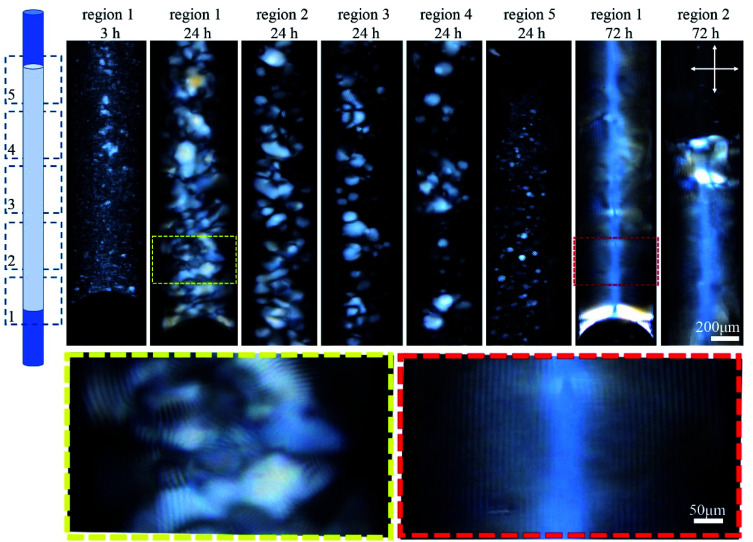
Time-series POM images of the 0.4 mm capillary tube filled with 4.5 wt% CNC, and aged vertically as shown in the cartoon. Images were collected along the length of the tube in five regions. Colored dashed boxes show high magnification images. Crossed arrows show the polarizer/analyzer configuration. Rotating the tube around its long axis, and imaging the bottom third shows the lines at different rotation angles implying complete filling of the bottom of the tube (see Fig. S2, ESI†).

The orientation of the tube during liquid crystal formation is also important in this low concentration regime of 4.5 wt%. In the experiments described in the previous paragraph, the tube was left in a horizontal orientation while the liquid crystal developed. When the tube was instead positioned vertically during liquid crystal formation, tactoid formation was again evident after 3 h (Fig. 3). After 24 h, larger tactoids had settled and fully filled the bottom of the tube, coalescing and producing a relatively disordered fingerprint texture. Moving toward the top of the tube, a progressive reduction in the size of the tactoids is clear, suggesting faster sedimentation for larger tactoids. No tactoids were observed after 72 h, and highly uniform lines were visible in the bottom third of the tube, above which a completely isotropic phase existed (Fig. 3).

**Fig. 4:**
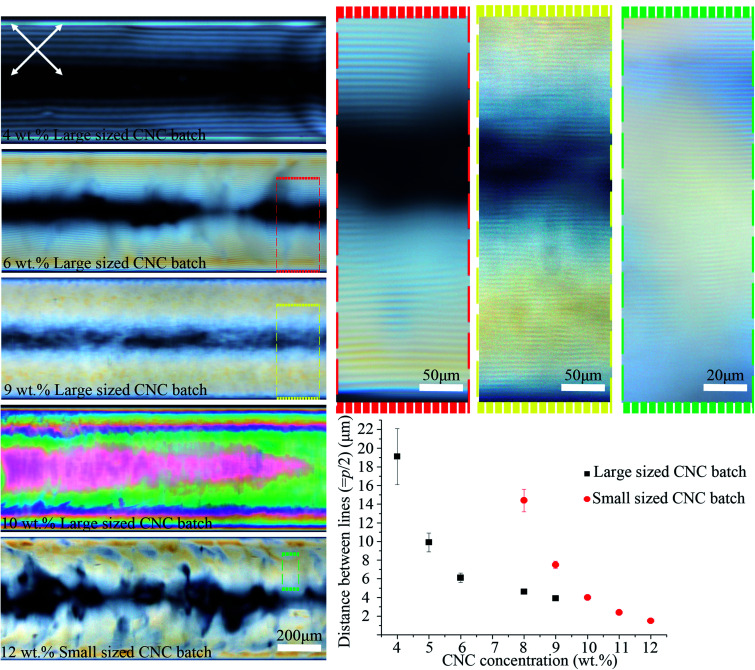
POM images of the 0.4 mm capillary tubes filled with different CNC concentrations and types. The average hydrodynamic radius of the particles for the large and small sized CNC batches were 193 ± 14 and 92 ± 4 nm, respectively. Capillary tubes were aged horizontally for the ordered structure to form. Colored dashed boxes show high magnification images. Crossed arrows show the polarizer/analyzer configuration. The graph plots the distance between periodic lines *vs.* [CNC] and type. Capillary tubes were placed at 45° between the polarizer and analyzer. The intensity of transmitted light depends on sin^2^(2*θ*), where *θ* is the angle between capillary axis and polarizer/analyzer. At diagonal orientation (*θ* = 45°), the intensity is maximum (see Video 1 and Fig. S9, ESI†).

As shown in Fig. 4, the distance between the periodic lines observed by POM decreased significantly as CNC concentration increased. The lines only appeared at CNC concentrations greater than *ca.* 4 wt%, and between 4–9 wt% the distance decreased from 19 ± 3 to 4 ± 0.2 μm. No lines were observed above *ca.* 9 wt% CNC concentration, but the sample looked colorful and highly birefringent by POM. At 10 wt%, the successive colors from the tube edge to its center follows the successive order of interference colors in the Michel-Levy chart^27^ and, as predicted, it ends in pink (the highest-order interference color) in the central region, where the sample is thickest. These results have been attributed to higher CNC interactions that enhance the intrinsic twist, resulting in a smaller pitch and agrees with the reported inverse relation between chiral nematic pitch and concentration in CNC suspensions.^28^ At high CNC concentration (>*ca.* 10 wt%), the high viscosity of the solution prevents liquid crystal formation as it limits the free motion of CNC particles.

We examined a second batch of CNCs with a smaller average particle size (92 ± 4 nm). We found that it behaved similarly, but the concentration at which structural order was observed increased to 8–12 wt% for the smaller sized CNC batch (Fig. 4) as opposed to 4–9 wt% for the larger one (193 ± 14 nm). The smallest distance between periodic lines observed in the smaller sized CNC batch was 1.5 ± 0.2 μm (Fig. 4), which occurred at 12 wt%. Previous reports have shown reducing the length of the CNC particles decreases the cholesteric pitch and increases the critical concentration required for cholesteric phase formation, in agreement with our result;^29,30^ however, the observed difference between CNC batches may not be due solely to the particles size,^31^ and instead could be caused by CNCs being obtained from different cellulose sources or due to variable reaction conditions during CNC extraction. The effect of salt addition was also tested by adding NaCl (0–12 mM) to a 9 wt% CNC suspension prior to capillary tube filling. Salt addition slightly reduced the distance between the lines from 4 ± 0.2 to 3 ± 0.3 μm (see Fig. S3, ESI†). At high salt concentration (12 mM), the liquid crystal phase was disrupted, and periodic lines were not observed by POM.

**Fig. 5:**
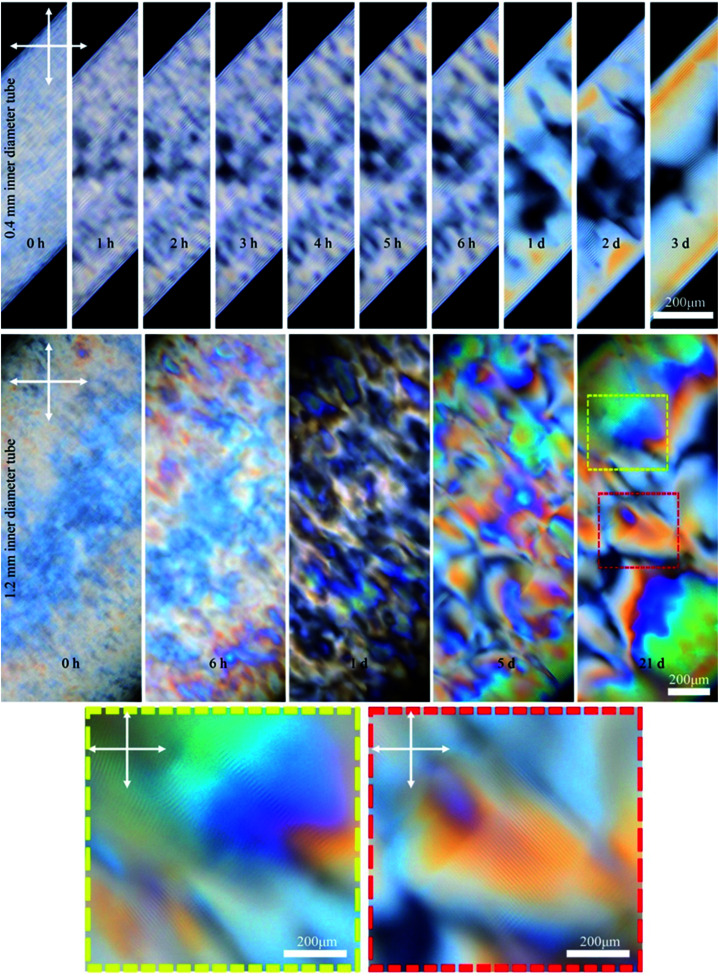
Time-series POM images of the 0.4 and 1.2 mm capillary tubes filled with 6 wt% CNC (larger sized CNC batch). Colored dashed boxes show high magnification images. Crossed arrows show the polarizer/analyzer configuration. Capillary tubes were placed at 45° between the polarizer and analyzer. Unlike the 4.5 wt% CNC that was isotropic at the beginning (Fig. 2), the 6 wt% CNC was highly birefringent at the beginning.

We investigated the effect of the capillary tube diameter on the liquid crystal structure by using tubes with 0.4 and 1.2 mm inner diameter. Fig. 5 shows time-series POM images of these tubes filled with 6 wt% CNCs. In the small (0.4 mm) tube, the fingerprint lines gradually appeared and the highly ordered single-domain structure formed within 3 days as exemplified by uniform birefringent color and evenly spaced parallel lines throughout the length of the tube (see also Fig. S4, ESI†). In contrast, CNCs confined in the large tubes (1.2 mm) did not form long-range order, even after 21 days. Instead, the sample showed a multidomain structure with birefringent colors and periodic lines with various orientations visible by POM in some regions of the tube (see Fig. 5 and also Fig. S5, ESI†). These findings suggest a small inner diameter of the capillary tube is important to obtaining a well-ordered monodomain structure of CNCs in the tube. It is the capillary tube glass surface that directs the CNC chiral nematic structure formation and, for the smaller tube, the higher surface to volume ratio helps with the uniform structure formation.

After understanding the evolution of CNC liquid crystalline domains inside the capillary tube, we sought to capture this structure in a polymer matrix. There are previous reports of capturing CNC structure through polymerization techniques such as photopolymerization of magnetically aligned CNCs in hydroxyethyl methacrylate (HEMA) or polyacrylamide hydrogels.^32,33^ Here we used hydroxyethyl acrylate (HEA) instead to obtain an elastic fiber. The tube was filled with a mixture containing CNCs and HEA monomer, *N*,*N*′-methylenebisacrylamide crosslinker, and 2-hydroxy-4′-(2-hydroxyethoxy)-2-methylpropiophenone initiator. After the sample aged horizontally for 72 h to form the ordered structure, polymerization of the organic monomers was initiated with UV irradiation, converting the HEA and crosslinker into poly(hydroxyethyl acrylate), PHEA. The resulting CNC/PHEA fiber could be easily removed from the capillary tube using a small needle (see Video 2, ESI†). The fiber has high water content; when a 6 wt% CNC solution is used, the resulting fiber has ∼85 wt% water, 9 wt% PHEA, and ∼6 wt% CNC.

**Fig. 6:**
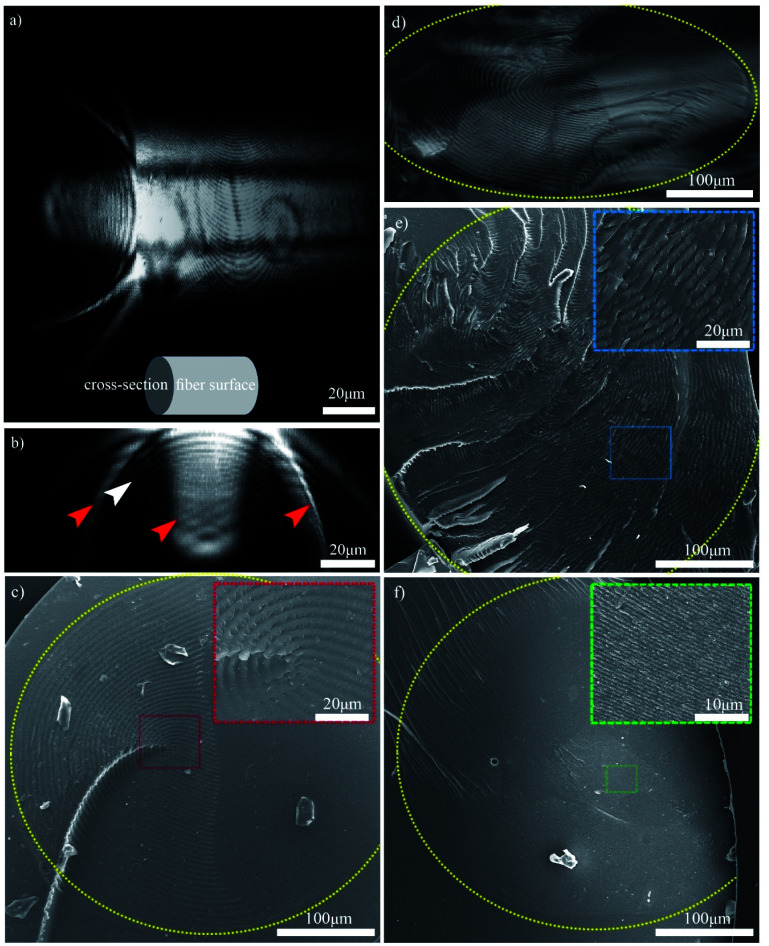
(a) 3D confocal reconstruction of the 6 wt% CNC/PHEA fiber showing its surface and cross-section; (b) confocal cross-section image of the 6 wt% CNC/PHEA fiber (red arrows indicate possible artifacts and the white arrow points to concentric rings); (c) SEM image of the 6 wt% CNC/PHEA fiber; (d) reflected light microscopy image of the 6 wt% CNC/PHEA fiber; (e) SEM image of the 9 wt% CNC/PHEA fiber; (f) SEM image of the 12 wt% CNC/PHEA fiber. Yellow colored dashed ovals highlight the fibers' circumference. Colored dashed boxes are insets showing higher magnification images of select regions. Larger sized CNC batch was used for the 6 and 9 wt% fibers while the smaller sized CNC batch was used for the 12 wt% fiber.

The 3D structure of the CNC/PHEA composite fiber was studied using the scattering mode of laser scanning confocal microscopy (LSCM). Fig. 6a shows the 3D structure of a CNC/PHEA fiber reconstructed from LSCM data. Videos showing the complete 3D structure, Z-stacks, and cross-sections are provided in the ESI (Videos 3–5†). The 3D structure and cross-section images clearly show concentric rings spanning the length of the fiber (Fig. 6a and b). In Fig. 6b, the intensity of scattered light from the concentric rings is maximum at the top of the fiber and gradually fades toward the center of the fiber. The other half of the fiber was completely dark (not shown in the image), suggesting an absence of ordered structure there. Observed scattering artefacts (indicated by red arrows in Fig. 6b) are possibly caused by nonlocal out-of-focus backscattering at these locations, a well-known issue with the scattering mode of LSCM.^34^ Presumably, the concentric rings (the white arrow in Fig. 6b) represent half helical pitches of the chiral nematic structure of the CNCs inside the fiber, which was further investigated by carrying out light microscopy and SEM imaging on the cross-section of the fibers (Fig. 6c–f). Remarkably, evenly spaced concentric rings were observed. For the CNC/PHEA fiber prepared using low CNC concentrations, the concentric rings observed are uniform and nearly defect-free, but they do not cover the whole cross-section (Fig. 6c and d). In contrast, at higher CNC concentrations, the concentric rings cover the whole cross-section (Fig. 6e), but more defects are apparent. The light microscopy image of the high CNC concentration fiber is provided in Fig. S6, ESI.† At high concentration, the distance between the concentric rings (*i.e.*, pitch) is smaller. The smallest pitch was observed at the highest CNC concentration (Fig. 6f), in agreement with POM images (Fig. 4), but there are some regions without the fingerprint texture. Distances between the periodic lines in POM imaging, and concentric rings in SEM and confocal microscopy are in good agreement (Table S1, ESI†).

Overall, based on these observations, a concentric cylindrical chiral nematic structure is proposed for the CNC/PHEA hybrid fiber as illustrated in Fig. 1b. The cholesteric axis is radial, going from fiber edge to its center. This type of structure is known as a radially twisted-axial configuration, or double twist cylinder geometry, and was previously reported for chiral nematic liquid crystals inside capillary tubes,^35–37^ but has not been captured using photopolymerization. There are two requirements for formation of this structure:^36^ (1) a cylinder with diameter larger than the intrinsic cholesteric pitch of the liquid crystal; and (2) parallel anchoring of the liquid crystal mesogens to the inner cylinder surface. In our case, both conditions are met: the CNC pitch is at least an order of magnitude smaller than the tube diameter, and CNC particles anchor parallel to the hydrophilic glass substrate.^38^

The previous report on confinement of CNCs inside a thin rectangular capillary tube has suggested a very different chiral nematic structure with cholesteric axis parallel to the length of the capillary tube.^23^ In contrast, the chiral nematic structure of our fibers is concentric showing a radial cholesteric axis. This discrepancy originates from a very different approach taken in the reference,^23^ where structure formation was induced through evaporation. In that reference, unlike our work, the capillary tube was not sealed. This led to evaporation from the capillary tube end which induced flow in that direction. CNCs were transferred to the evaporation boundary where they formed a planar chiral nematic structure. In that structure, the cholesteric axis was parallel to the capillary length because the nucleation site was not the glass capillary surface, but instead it was the air–liquid interface at the evaporation edge. In our case, the nucleation site is the glass capillary surface, which in combination with the round geometry of our capillary tubes, led to the observed concentric chiral nematic structure.

**Fig. 7:**
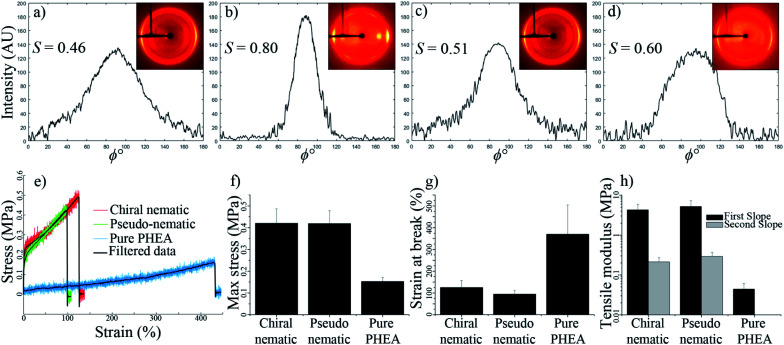
2D-XRD profile and intensity values for the (200) plane (2*θ* = 22.9°) along the azimuthal angle (*ϕ*); (a) pseudo-nematic CNC/PHEA fiber, (b) pseudo-nematic CNC/PHEA fiber stretched 2×, (c) chiral nematic CNC/PHEA fiber, and (d) chiral nematic CNC/PHEA fiber stretched 2×. Hermans order parameter (*S*) is also reported (error estimate for reported *S* values is ±0.01). Tensile properties of the fibers; (e) raw and filtered strain–stress curves, (f) maximum stress at break, (g) strain at break, (h) tensile moduli values.

Fig. 7a and b show the 2D-XRD measurements of a CNC/PHEA fiber that was polymerized immediately after filling the tube, where the CNCs did not have time to organize into a chiral nematic liquid crystal. Data are shown for the sample in a relaxed condition and during 2× stretching parallel to the long axis. Diffraction intensities *I*(*ϕ*) with respect to the azimuthal angle (*ϕ*) at (200) plane (2*θ* = 22.9°) are maximum at the equatorial position, indicating CNC alignment parallel to the fiber long axis. The alignment improves upon stretching, with an increase of the Hermans order parameter (*S*) from 0.46 to 0.80. This suggests that the shear force during the filling of the capillary tube caused the CNC spindles to partially align parallel to the length of the tube and stretching improved the alignment, effectively transforming them from a pseudo-nematic to a nematic arrangement. *S* values close to 0.8 have only been reported for highly aligned CNC particles obtained at high shear forces.^39^

Next, a capillary tube was filled with CNCs and the PHEA precursors, and was polymerized after 72 h of horizontal aging, allowing the concentric structure to form. Fig. 7c and d show the 2D-XRD pattern of this fiber while relaxed and during 2× stretching, respectively. Similar to the pseudo-nematic sample, the (200) diffraction ring of this chiral nematic structure shows high intensity at equatorial positions, giving *S* values of 0.51 and 0.60 for relaxed and stretched fibers, respectively. Fibers kept their chiral nematic structure after stretching and the chiral nematic structure did not unwind into a nematic structure. This was also confirmed by the persistence of the fingerprint lines in the fiber at different extents of stretching (see Fig. S7, ESI†). Numerical simulations of chiral nematic liquid crystals inside capillary tubes have predicted a value of ∼0.6 for a perfectly concentric chiral nematic structure.^37^

The CNC/PHEA fibers are highly stretchable (see Video 6, ESI†). Tensile tests were carried out on the concentric chiral nematic CNC/PHEA fiber and compared to the pseudo-nematic CNC/PHEA fiber and a pure PHEA fiber (without CNCs). Fig. 7e shows the stress–strain curves for the three fiber types. Interestingly, mechanical properties of the chiral nematic and the pseudo-nematic fibers are very similar (Fig. 7f–h). This is unexpected as in the pseudo-nematic fiber the CNC particles are mostly aligned parallel to the fiber length (the direction of tension), but in the chiral nematic fiber the director rotates, and the CNC particles are not generally aligned parallel to the direction of tension. Both fibers have similar tensile moduli and high strength, but they are not as stretchable as the pure PHEA fiber. Addition of CNCs clearly has a reinforcing effect on the polymer, but it also decreases elasticity. In contrast to the pure PHEA fiber, CNC/PHEA composite fibers underwent a permanent plastic deformation and did not rebound to their original shape upon release after stretching (see Videos 6 and 7, ESI†).

The structure of the single-domain chiral nematic concentric CNC/PHEA fiber is very similar to biological hierarchical structures, such as the twisted plywood architecture of collagen fibers in the osteon of cortical bone lamellae.^40^ In wood and plant cells walls, cellulose microfibrils wind around the hollow core, where cells reside, forming multiple concentric layers, known as wood cell units.^2^ We showed that polymerization of a liquid crystal system confined inside a capillary tube could achieve similarly complex structures. Many applications could be envisioned based on the CNC fiber platform. Fibers could be bundled or weaved to develop larger, higher dimensional structures with multiple levels of hierarchy. These fibers could also be used as a template to develop other concentric chiral nematic organic or inorganic materials. An interesting application of CNCs is in the field of photonics. Chiral nematic structures reflect circularly polarized light when the wavelength matches their helical pitch.^18^ One of our goals was to prepare stretchable CNC/PHEA fibers that show structural color upon stretching, but the smallest pitch we obtained was ∼3 μm – far from the required sub-micron range (Fig. 4). Both drying and stretching decrease the fiber diameter and consequently decrease the pitch value to the same extent. Fig. S7 and S8 (ESI†) show the decrease of the distance between fingerprint lines after stretching and drying. The distance between the layers decreases upon drying or stretching the fiber, and consequently the pitch decreases. For our smallest pitch fiber, a ∼4× reduction in the fiber diameter is required to bring the pitch into sub-micron range. After drying and stretching, however, this level of fiber thinning was not feasible, and we could not observe structural color from the fibers. We are currently exploring modification of the polymer fiber composition and using CNCs from other sources that have a smaller aspect ratio (and pitch) to develop fibers with pitch closer to this sub-micron range. Another possible application for the concentric CNC fibers is in the field of fiber optics. Existing optical fibers rely on total internal reflection or diffraction from a periodic structure. A concentric chiral nematic CNC optical fiber would rely on a completely different phenomenon. It should be possible to transfer left-handed circularly polarized light with a wavelength matching the CNC fiber's pitch. Depending on the transmitted light wavelength, a fiber of desired pitch could be designed, and the inner core could be hollow. This approach may lead to optical fibers covering a large range of wavelengths, high flexibility, and minimal manufacturing cost. In practice, translating these fibers into optically applicable fibers faces several challenges, including controlling the pitch size and the fiber length, and developing a uniform distribution of chiral nematic structure along the fiber circumference instead of a fiber structure with only half of the cholesteric liquid crystal phase.

## Conclusion

Chiral nematic CNC polymer fibers with controllable pitch were fabricated. We combined capillary confinement, photopolymerization and CNC liquid crystals to develop these uniquely ordered fibers. We evaluated a series of variables to understand and control the self-assembly behavior of CNCs in capillary confinement. Through photopolymerization, the structure of the chiral-nematic phase was locked to obtain chiral nematic fibers, which were investigated with respect to their structural features as well as their mechanical properties. In this work, we tried to understand and demonstrate the feasibility of forming these highly ordered fibers and to understand the limitations and challenges. Addressing these challenges would bring us closer to applying these fibers to the field of optics.

## Experimental

### Materials

Aqueous suspensions of CNCs prepared from sulfuric acid hydrolysis of Kraft softwood pulp were supplied by FPInnovations (4 and 6 wt%, acid form of CNCs with pH = 2.0). Two types of CNCs were used; a larger sized CNC batch with an average hydrodynamic radius of 193 ± 14 nm (*n* = 18) and zeta potential of −51.2 ± 2.3 mV (*n* = 7) (measured using a Brookhaven NanoBrook Omni Dynamic Light Scattering instrument), and a smaller sized CNC batch with an average hydrodynamic radius of 92 ± 4 nm (*n* = 18) and zeta potential of −49.2 ± 2.2 mV (*n* = 7). Experiments described throughout the paper used the larger sized CNC batch unless it is explicitly specified that the smaller size was used. Higher concentrations of CNC suspension were prepared from the original 4 or 6 wt% CNC suspensions using rotary evaporation. The CNC concentration was confirmed by measuring the weight of a known volume of CNC suspension after drying in triplicate. Hydroxyethyl acrylate (HEA) (Aldrich, 98%), hydroxyethyl methacrylate (HEMA) (Aldrich, 98%), *N*,*N*′-methylenebisacrylamide (Aldrich, 99%), and 2-hydroxy-4′-(2-hydroxyethoxy)-2-methylpropiophenone (Aldrich, 98%) were used as received.

### Formation of CNC chiral nematic structure

Before filling the capillary tube, the CNC solution was bath-sonicated for 15 min. Silicate glass capillary tubes with inner diameter of 0.4 mm (7.6 cm in length, Drummond Scientific Co.) and 1.2 mm (Kimble Chase) were filled with CNC suspension based on capillary action. Alternatively, at concentrations higher than 9 wt%, the CNC suspension was injected into the capillary tube. The suspension inside the filled capillary tube was adjusted to leave air gaps at both ends of the tube. The tube ends were sealed using 5 min epoxy (Devcon), fixed on a glass slide, oriented horizontally or vertically, and aged to monitor formation of the chiral nematic structure. Different CNC concentrations and CNC types were tested. The effect of salt addition was tested by adding NaCl (0–12 mM) to a 9 wt% CNC suspension prior to capillary tube filling.

### Formation of CNC/PHEA polymeric fiber

1 g of HEA monomer, 20 mg of *N*,*N*′-methylenebisacrylamide (crosslinker), and 6 mg of 2-hydroxy-4′-(2-hydroxyethoxy)-2-methylpropiophenone (initiator) were dissolved in a 10 mL aqueous CNC suspension. This solution was used to fill the silicate glass capillary tubes as described above. Different CNC concentrations and CNC types were used. The filled capillary tubes were irradiated with a 300 nm ultraviolet-B-light source (8 W) for 1 h to complete the photopolymerization of the monomers. The polymerization was carried out either immediately after capillary tube filling, resulting in a pseudo-nematic CNC/PHEA fiber, or after 72 h aging, resulting in a chiral nematic CNC/PHEA fiber. The polymerized fiber was removed from the capillary tube using a LUTER 0.35 mm 3D printer nozzle cleaning needle.

### Polarized optical microscopy (POM) imaging

CNC filled capillary tubes and polymeric fibers were imaged between crossed polarizers using an Olympus BX53M microscope. Images were collected on a rotating stage using a white light source without a waveplate. Images were collected either at parallel or diagonal orientation of the tube with respect to polarizer/analyzer (see Fig. S9†). The periodic distance between lines (chiral nematic pitch) was measured by determining RGB intensity distribution along the fiber diameter using a custom algorithm developed in MATLAB® (MathWorks Inc.). The periodic lines in the POM images result in an RGB intensity distribution curve with an oscillating pattern (see Fig. S10, ESI†). The distance between the peaks in this oscillating pattern was measured for >20 replicates, reporting the average and standard deviation. The apparent inner diameter of the tubes in POM images is 0.55 mm while the actual inner diameter of the tube is 0.4 mm (see Fig. S1, ESI†). The true distance between the periodic birefringent lines is the apparent distance divided by 1.37 (=0.55/0.4), and this corrected value is reported throughout the paper.

### Scattering mode laser scanning confocal microscopy imaging

A 6 wt% CNC/PHEA fiber was removed from the capillary tube after polymerization, fixed flat on a glass slide and imaged using a Zeiss LSM 510 confocal microscope. The local scattering throughout the sample was imaged using scattering mode of the confocal microscope using a 63× objective lens. The sample was scanned on the *xy*-plane, and 53 stacks were collected along the *z*-axis, through the fiber thickness. The 3D reconstruction and videos were developed using ImageJ (NIH Image, Bethesda, MD). Maximum intensity cross-section projection showing concentric rings was used to measure the distance between the rings using a custom algorithm developed in MATLAB® (MathWorks Inc.) (see Fig. S11, ESI†).

### Scanning electron microscopy (SEM) imaging

SEM images were collected on the cross-section of the fibers. A HEMA solution was prepared by dissolving 7.4 mg of 2-hydroxy-4′-(2-hydroxyethoxy)-2-methylpropiophenone (initiator) in 2.14 g of HEMA. CNC/PHEA fibers with 0.4 mm diameter were removed from the capillary tube after polymerization, placed in a 1.4 mm capillary tube, filled with the HEMA solution, sealed, and left overnight for HEMA to replace water in the CNC/PHEA fiber. The contents of the capillary tube were photopolymerized as described before. The polymerized capillary tube was broken and the cross-sections were fixed on a stub, sputter coated using a Leica EM ACE600 (5.6 nm iridium) and imaged using FEI Nova NanoSEM430 with an accelerating voltage of 5–15 kV. Cross-sections were also imaged using light microscopy.

### 2D X-ray diffraction (2D-XRD) studies

The pseudo-nematic CNC/PHEA fiber and the chiral nematic CNC/PHEA fibers were removed from the capillary tubes, fixed using a paper clip, and 2D-XRD patterns collected. The 6 wt% (larger-sized CNC batch) was used to prepare the fibers. The fiber long axis was positioned vertically for the XRD (positioning the fiber horizontally only rotates the 2D-XRD pattern). It was necessary to remove the polymerized fibers from the glass tube otherwise the amorphous silicate diffraction would obscure the CNC diffraction peaks. Fibers were measured at both a relaxed and a 2× stretching position. Images were recorded on a Bruker APEX DUO with APEX II CCD detector using Cu Kα_1_ X-ray beam with a wavelength of 0.154 nm at 0.6 mA, 45 kV for 480 s at 40 and 60 mm from the detector in transmission mode. Multiple rings are visible corresponding to X-ray diffraction of different cellulose crystal lattices with the brightest diffraction ring at 2*θ* = 22.9° corresponding to the (200) diffraction of the cellulose Iβ crystal,^41^ where cellulose polymer chains in a single CNC are aligned parallel to the CNC rod length.^39^ Diffraction intensities *I*(*ϕ*) with respect to the azimuthal angle *ϕ* were determined at 2*θ* = 22.9° by fitting a circle on the corresponding ring, and measuring pixel intensity along the circumference. The north pole of the ring corresponds to *ϕ* = 0° in the measurements. Hermans order parameter (*S*) was calculated from the (*ϕ*, *I*(*ϕ*)) values using eqn (1) and (2),^39,42^ through a numerical analysis in MATLAB® (MathWorks Inc.), after subtracting the background. *S* can range between 0 and 1, with 0 indicating an isotropic structure and 1 indicating a perfectly aligned structure.1
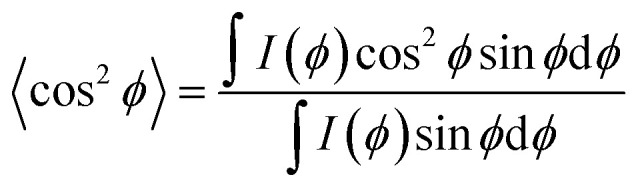

2 *S* = 1 − 3〈cos^2^ *ϕ*〉

### Tensile mechanical testing

When CNC/PHEA fibers were initially removed from the capillary tube, they had high water content and, despite being sturdy and flexible, they were not stretchable. PHEA with high water content acts more like a hydrogel and lacks any stress-releasing mechanism, leading it to break under tension.^43^ A completely dry fiber is also not suitable as it is brittle, especially at high CNC content. This is likely due to strong hydrogen bonding between CNC particles.^44^ The ideal fiber should retain some water or contain a plasticizer. To obtain fibers with good mechanical properties, glycerol was added; glycerol is commonly applied in industry to prevent cracks in cellulose-based dialysis tubes.^45^ After polymerization, fibers were removed from the capillary tubes and placed in a 2.5 v% glycerol solution for 1 h and then dried. Glycerol remains as a plasticizer and prevents complete drying due to its hygroscopic nature. The fiber has ∼35 wt% CNC, ∼35 wt% PHEA, ∼15 wt% glycerol and ∼15 wt% water. Tensile testing was performed on 9 wt% (larger sized CNC batch) pseudo-nematic CNC/PHEA, chiral nematic CNC/PHEA fiber, and pure PHEA (no CNCs) fibers using a 5960 Series Universal Testing System (Instron) equipped with a 50 N load cell. The two ends of the fiber were glued to square shaped Teflon pieces, and positioned between the grips. The strain rate was 5 mm min^−1^ and seven replicates were measured for each fiber. Data were smoothed using a low pass filter with cut-off frequency of 60 Hz in MATLAB® (MathWorks Inc.). The smooth curves were used to determine the maximum stress-at-break, strain-at-break, and tensile modulus. Tensile modulus is reported by measuring the slope of the stress *vs.* strain curve. The cross-section area of the fibers was determined using light microscopy.

## Author contributions

A. M. is credited for the conception, design, analysis and writing of the study. C. M. W. is credited for carrying out the mechanical tests and authorship. Y. T. X. is credited for the conception and authorship. W. Y. H. is credited for providing CNC and authorship. M. J. M. is credited for the conception, design, supervising and authorship.

## Conflicts of interest

There are no conflicts to declare.

## Supplementary Material

NA-003-D1NA00425E-s001

NA-003-D1NA00425E-s002

NA-003-D1NA00425E-s003

NA-003-D1NA00425E-s004

NA-003-D1NA00425E-s005

NA-003-D1NA00425E-s006

NA-003-D1NA00425E-s007

NA-003-D1NA00425E-s008
