# Supplementary material for: Concentric chiral nematic polymeric fibers from cellulose nanocrystals
Source: Nanoscale Adv. 2021 Aug 11;3(17):5111–21. doi: 10.1039/d1na00425e (PMC9416860; doi:10.1039/d1na00425e)
Supplement: NA-003-D1NA00425E-s001 [file NA-003-D1NA00425E-s001.pdf]

Electronic Supplementary Material (ESI) for Nanoscale Advances.
This journal is © The Royal Society of Chemistry 2021

Supporting Information

Concentric Chiral Nematic Polymeric Fibers from Cellulose Nanocrystals

Arash Momeni [a], Christopher M. Walters [a], Yi-Tao Xu [a], Wadood Y. Hamad [b], Mark J. MacLachlan* [a,c,d,e]

[a] Department of Chemistry, University of British Columbia, 2036 Main Mall, Vancouver, British Columbia V6T 1Z1, Canada
[b] Transformation and Interfaces Group, Bioproducts Innovation Centre of Excellence, FPInnovations, 2665 East Mall, Vancouver, British Columbia V6T 1Z4, Canada
[c] Stewart Blusson Quantum Matter Institute, 2355 East Mall, Vancouver, British Columbia, V6T 1Z4, Canada.
[d] WPI Nano Life Science Institute, Kanazawa University, Kanazawa, 920-1192, Japan.
[e] UBC BioProducts Institute, 2385 East Mall, Vancouver, British Columbia, V6T 1Z4, Canada

Supporting information videos:

Video 1. Polarized optical microscopy video shows a capillary tube filled with 6 wt.% CNC and the microscope stage as it is being rotated from 45° to 0° between the polarizer and analyzer. At 45°, the sample appears highly birefringent.

Video 2. Removal of a CNC/PHEA fiber from the capillary tube using a small needle.

Video 3. 3D structure of a 6 wt.% CNC/PHEA fiber imaged using scattering mode of laser scanning confocal microscopy.

Video 4. Z-stacks of a 6 wt.% CNC/PHEA fiber imaged using scattering mode of laser scanning confocal microscopy.

Video 5. Cross sections of a 6 wt.% CNC/PHEA fiber imaged using scattering mode of laser scanning confocal microscopy.

Video 6. Stretching CNC/PHEA fiber.

Video 7. Stretching pure PHEA fiber.

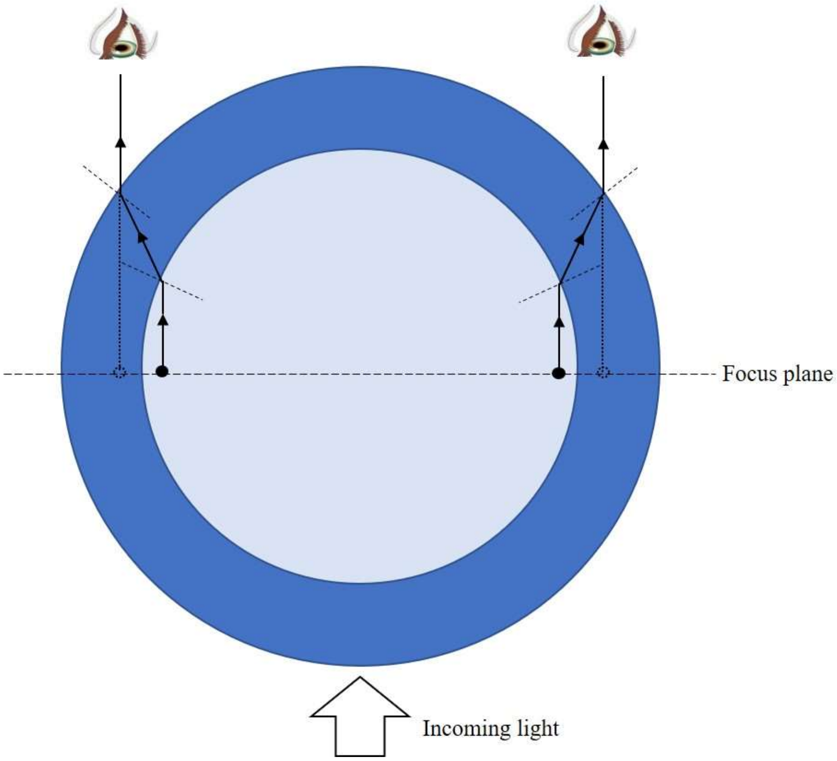

Figure S1. Schematic representation of the refraction path of light through a cylindrical capillary tube. Dark blue represents capillary tube thickness and light blue represents the liquid inside the tube. The light refraction causes the apparent inner diameter of the capillary tube in microscopy imaging to be larger than the actual inner diameter.

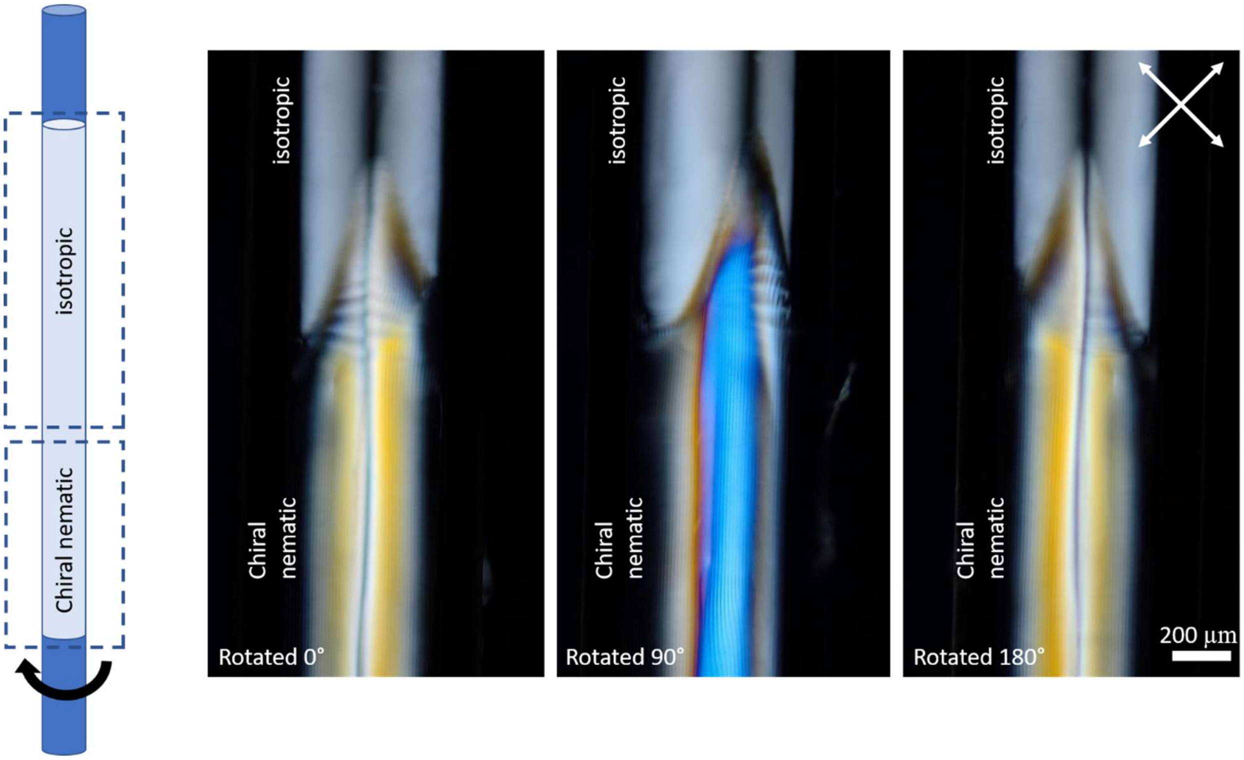

Figure S2. POM images of the 0.4 mm capillary tube filled with 4.5 wt.% CNC, aged vertically as schematically shown. Images are collected from the region of the tube where sample transit from chiral nematic to isotropic phase, at three rotation angles. Rotating the tube around its long axis, as shown schematically, shows the birefringent lines at different rotation angles implying complete filling of the bottom of the tube. The sample was locked in place using UV-initiated polymerization before POM imaging. Cross arrows show the polarizer/analyzer configuration.

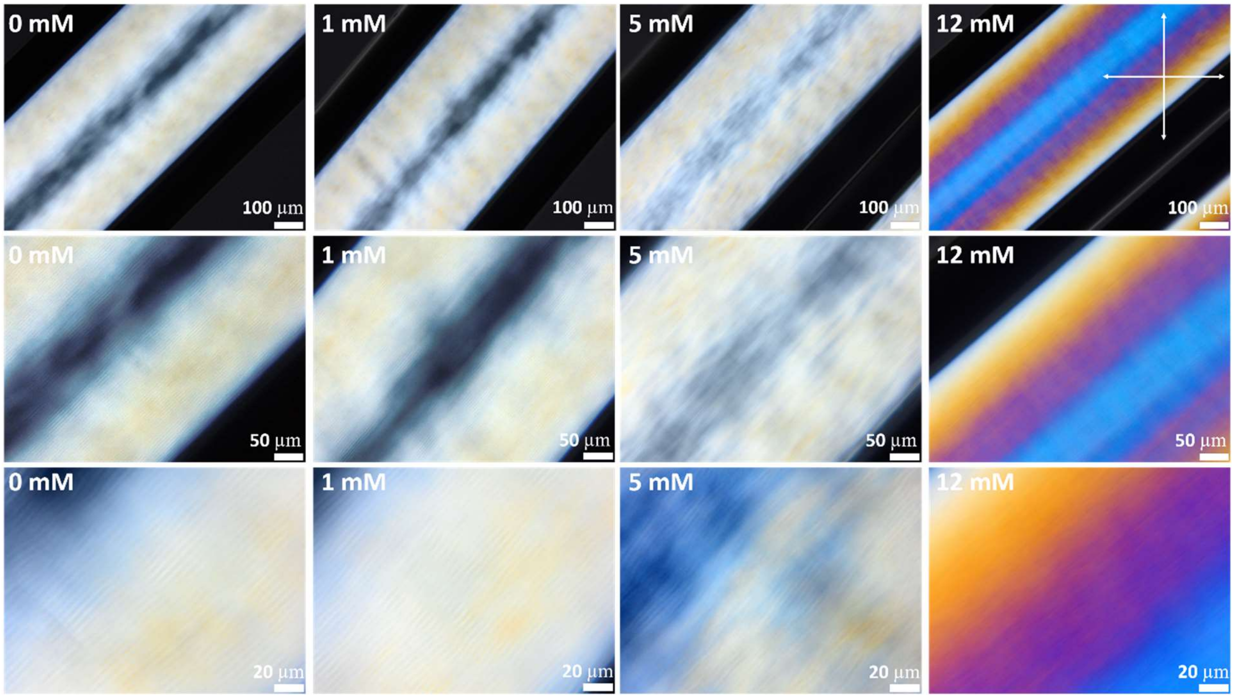

Figure S3. The effect of salt (NaCl) addition to a 9 wt.% CNC suspension prior to capillary tube filling. POM images of the filled capillary tubes are collected after 72 h, at different salt concentrations, and different magnifications. The scale bar for images on the top, middle, and bottom rows are 100, 50, and 20 μm, respectively. Salt addition slightly decreased the distance between periodic birefringent lines, however, high salt concentration (12 mM) disrupted the liquid crystal phase formation, and the periodic lines were not observed. Cross arrows show the polarizer/analyzer configuration.

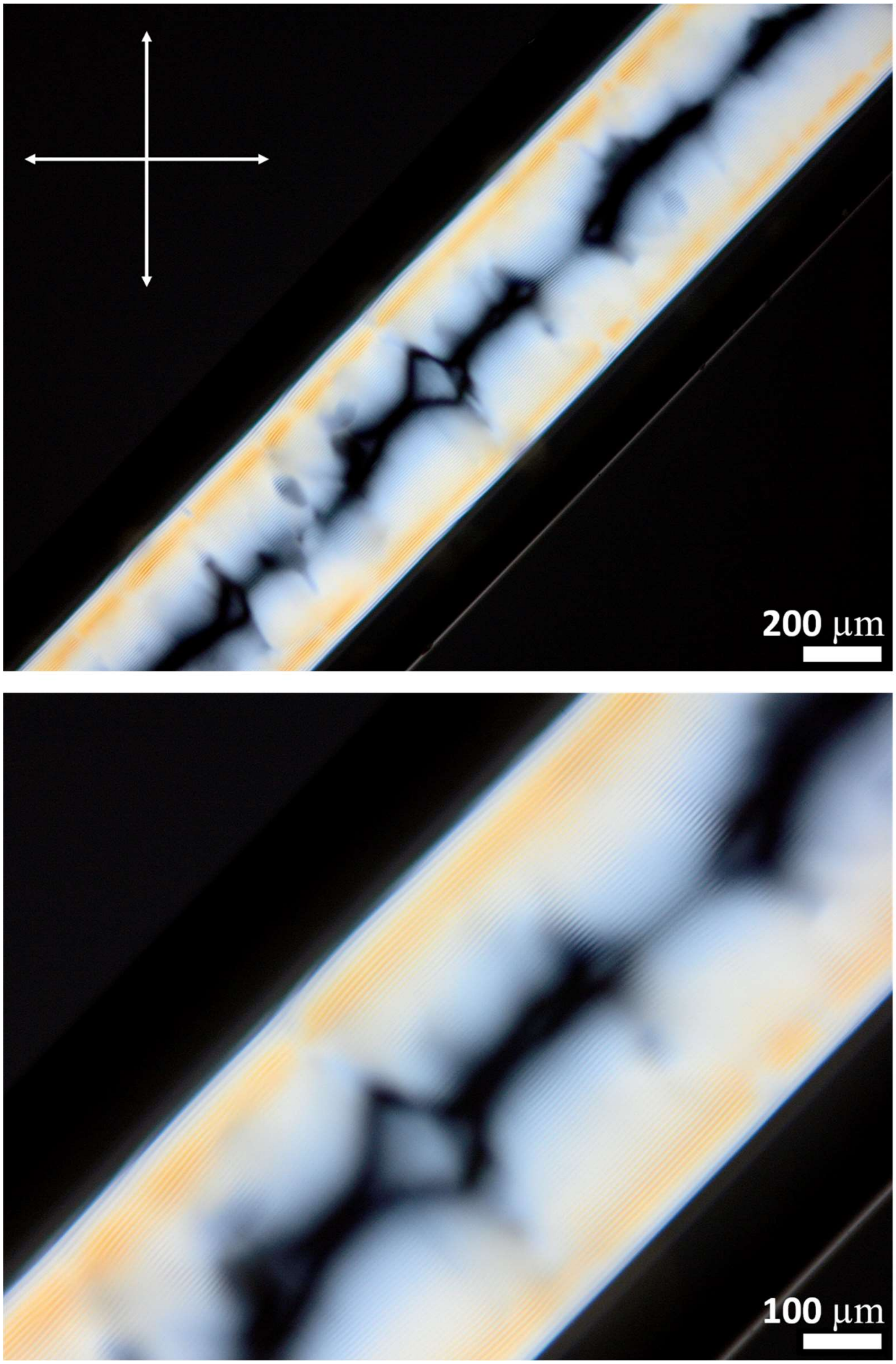

Figure S4. POM images of a capillary tube with inner diameter of 0.4 mm filled with 6 wt.% CNC suspension and aged 3 days. POM images are collected at two magnification. The scale bar for the top and bottom image is 200 and 100 µm, respectively. Cross arrows show the polarizer/analyzer configuration.

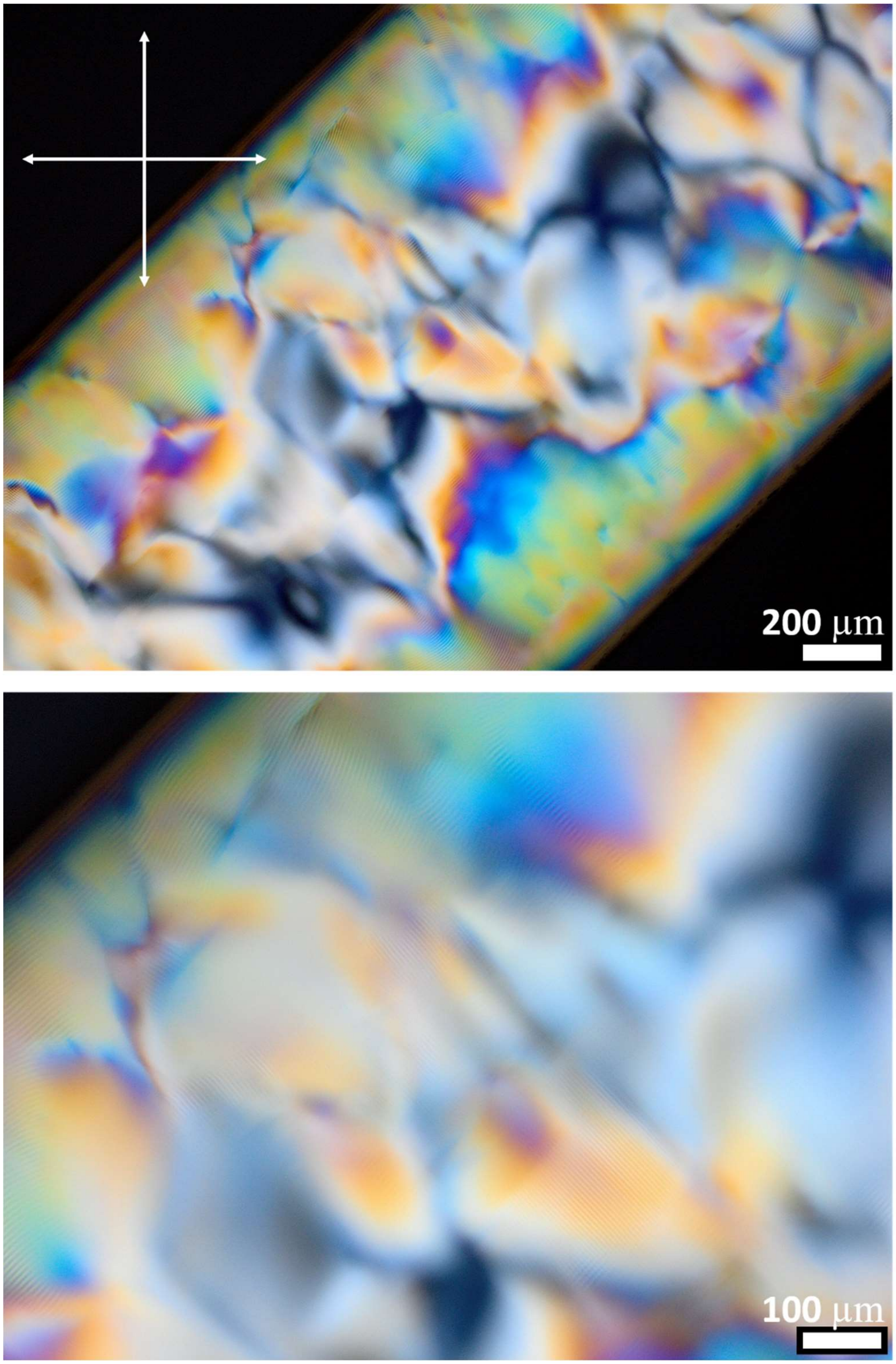

Figure S5. POM images of a capillary tube with inner diameter of 1.2 mm filled with 6 wt.% CNC suspension and aged 21 days. POM images are collected at two-fold magnification. The scale bar for the top and bottom image is 200 and 100 µm, respectively. Cross arrows show the polarizer/analyzer configuration.

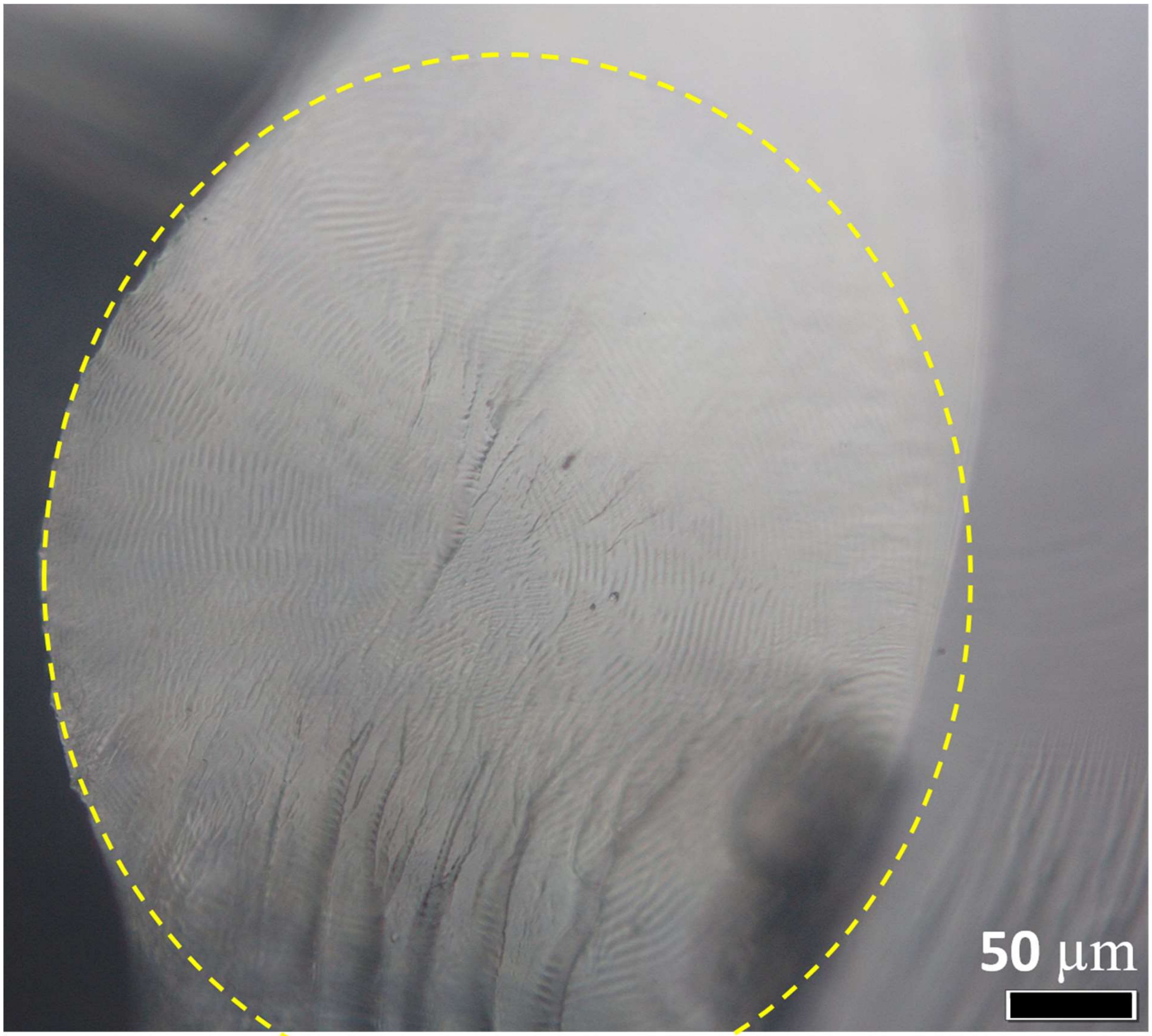

Figure S6. Reflected light microscopy image of the 9 wt.% CNC/PHEA fiber. Yellow colored dashed oval highlights the fiber's circumference.

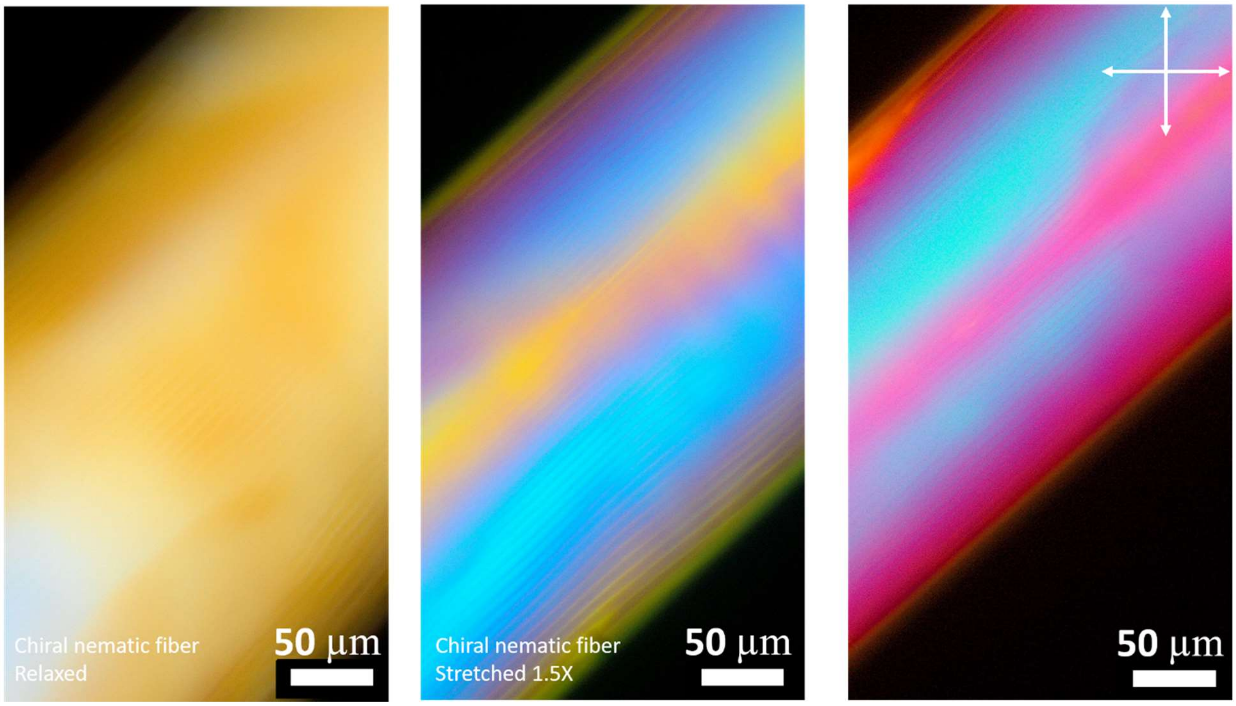

Figure S7. POM images of a CNC/PHEA fiber while relaxed and stretched 1.5x and 2x. Periodic birefringent lines in the fiber persist at different levels of stretching. Stretching decreases the fiber diameter and consequently decreases the distance between the periodic birefringent lines to the same extent. The distance between the periodic birefringent lines at relaxed, 1.5x and 2x stretching is 7±0.7, 6±0.6 and 5±0.2 μm, respectively. The scale bar for images is 50 μm. Cross arrows show the polarizer/analyzer configuration.

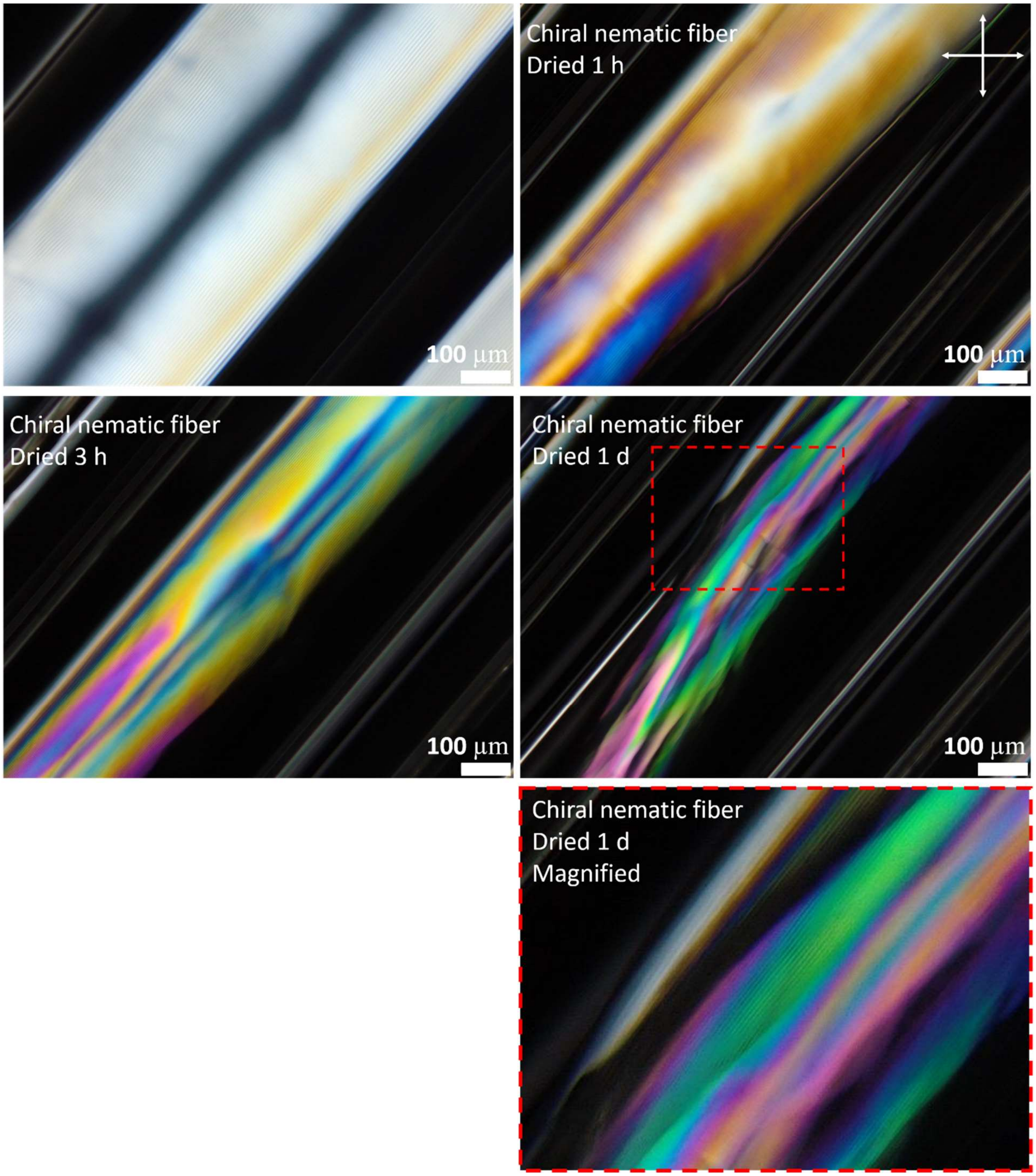

Figure S8. POM images of a CNC/PHEA fiber during drying process. Periodic birefringent lines in the fiber persist at different levels of drying. Drying decreases the fiber diameter and consequently decreases the distance between the periodic birefringent lines to the same extent. The distance between the periodic birefringent lines in the wet state is 8±0.4 µm, but after complete drying (1 day) is 3±0.4 µm. The scale bar for images is 100 µm. Cross arrows show the polarizer/analyzer configuration.

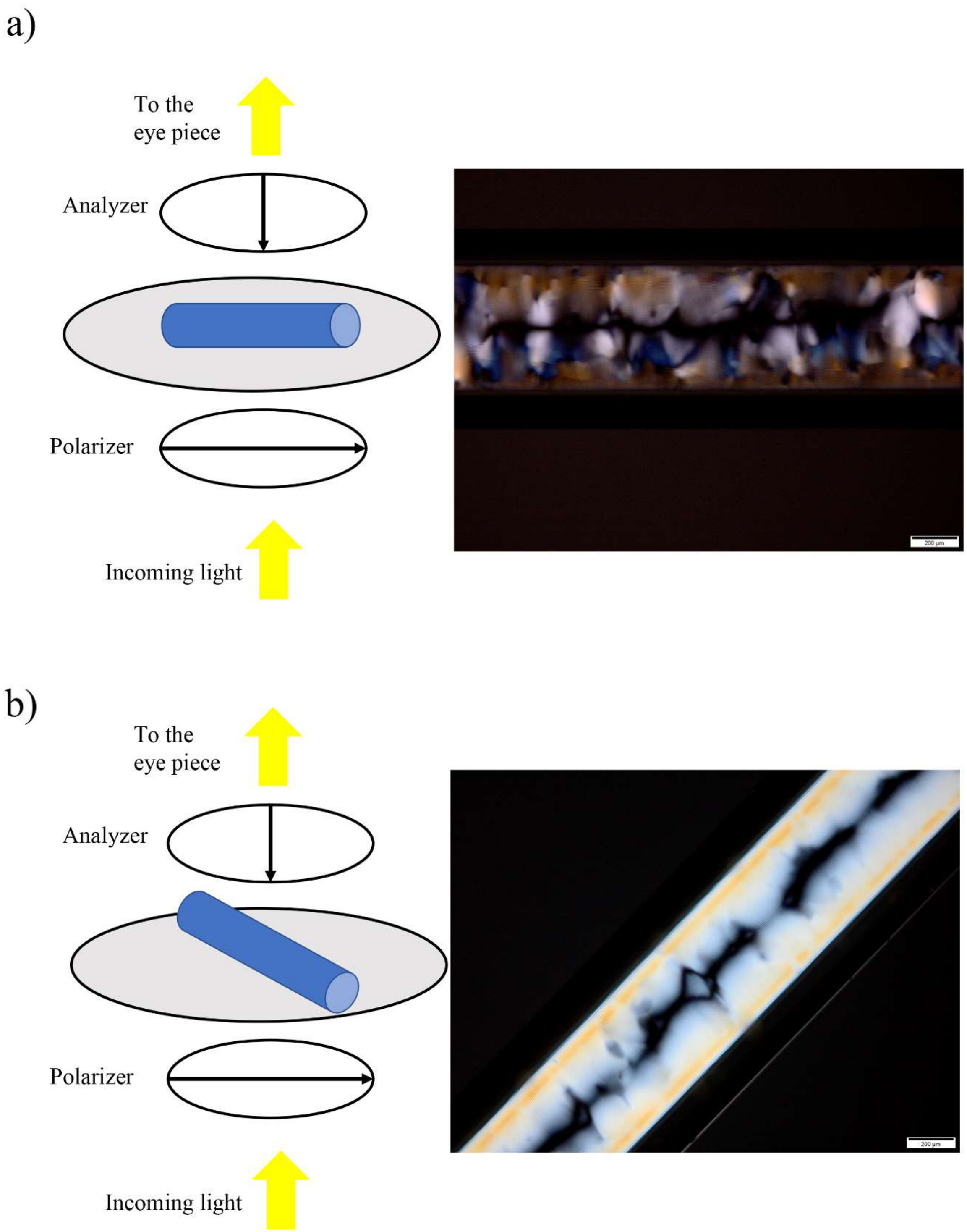

Figure S9. Schematic of the POM technique used in this study. The tube is positioned either at 0° (a) or 45° (b) between polarizer and analyzer. Corresponding images the tube viewed under these conditions are also provided. The image is very intense at diagonal position (b) compared to the dark image obtained at parallel position (a). In POM imaging, the intensity of transmitted light depends on $sin^2(2\theta)$, where θ is the angle between optical axis and polarizer/analyzer. At parallel orientation (θ = 0°), nearly no light passes, but at diagonal orientation (θ = 45°), the light intensity is maximum.

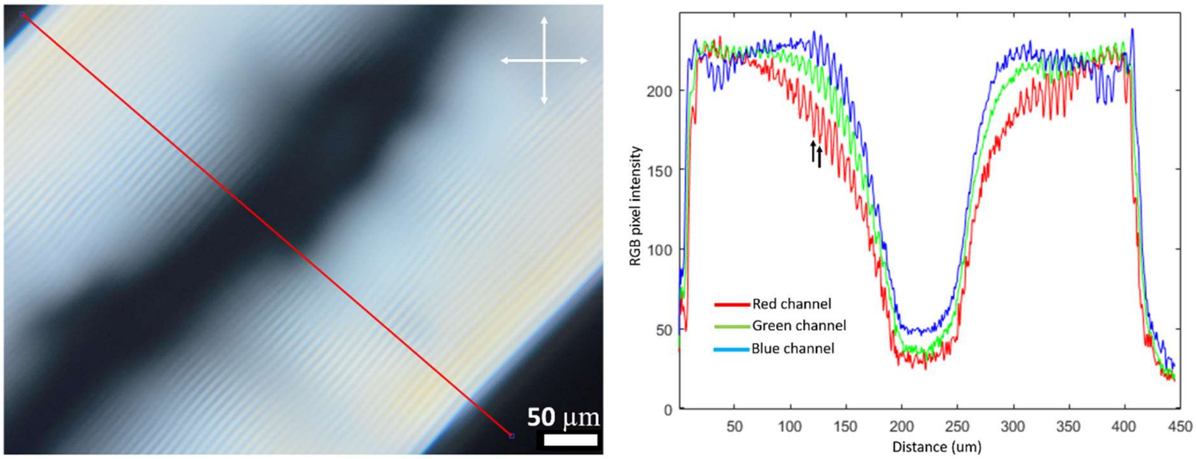

Figure S10. Measurement of the distance between the periodic birefringent lines by plotting an RGB intensity distribution curve. A line is drawn over the POM image (the red line) and the RGB intensity distribution curve along this line is plotted resulting in an oscillating pattern. The distance between the peaks in this oscillating pattern (shown by back arrows) is equal to half a pitch. POM image is of a 6 wt.% CNC suspension inside a 0.4 mm capillary tube. The scale bar for images is 50 μm. Cross arrows show the polarizer/analyzer configuration.

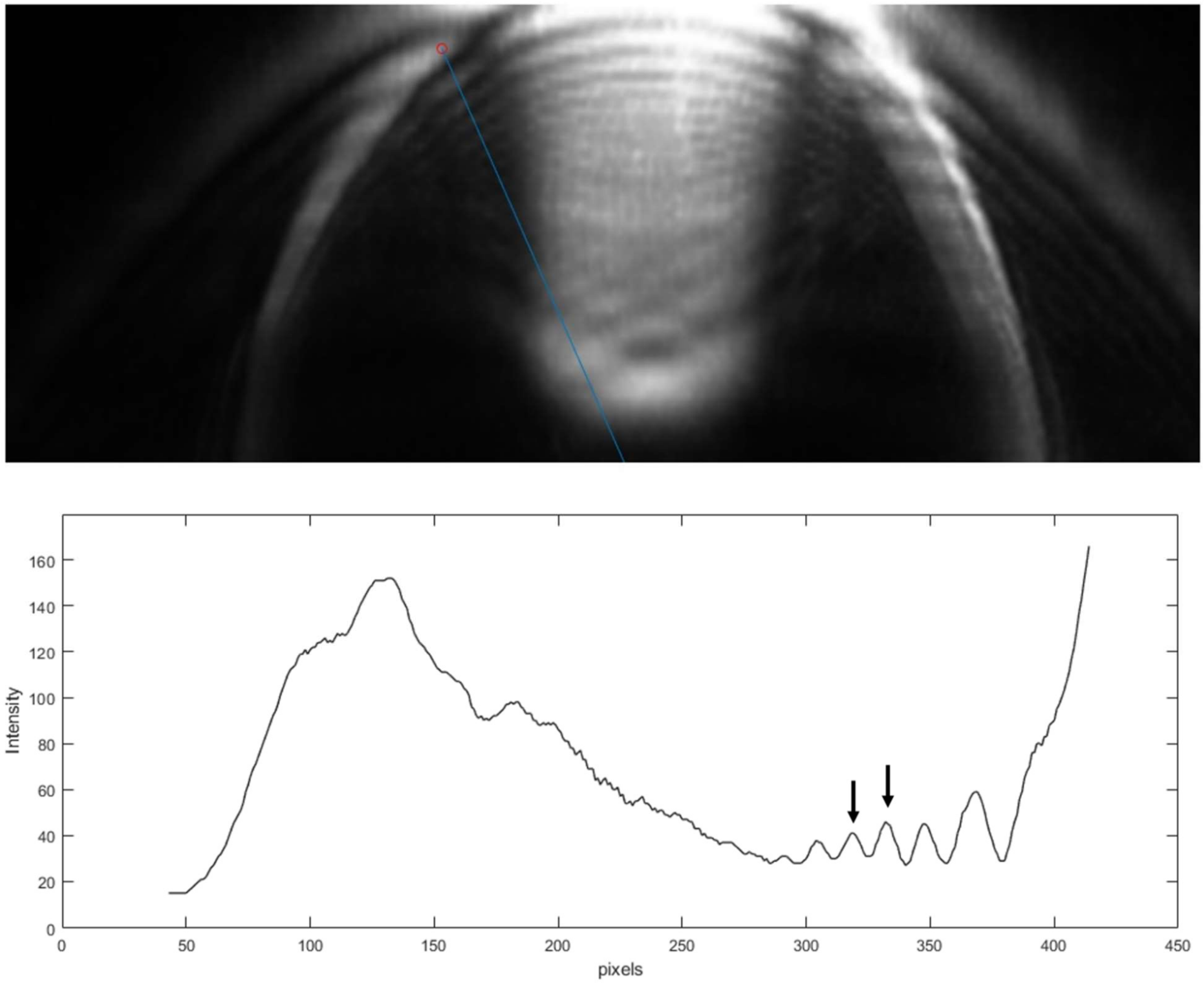

Figure S11. Measurement of the distance between the concentric rings in a cross section of a CNC/PHEA fiber. A line is drawn over the LSCM image (the blue line) and the gray scale intensity distribution curve along this line is plotted resulting in an oscillating pattern. The distance between the peaks in this oscillating pattern (shown by back arrows) is equal to half a pitch. LSCM image is of a dried 6 wt.% CNC/PHEA fiber.

Table S1. Distance between the periodic lines in POM imaging, and concentric rings in SEM and confocal microscopy.

Sample	Distance between the periodic lines in POM (μm)	Distance between the concentric rings in confocal (μm)	Distance between the concentric rings in SEM (μm)
6 wt.% CNC/PHEA fiber	6 ± 0.5	6 ± 1[a]	5.8 ± 0.8
9 wt.% CNC/PHEA fiber	4 ± 0.2	NA	3.8 ± 0.4
12 wt.% CNC/PHEA fiber [b]	1.5 ± 0.2	NA	1.0 ± 0.1

[a] This value is calculated by measuring the distance between the concentric rings in the confocal image × (fiber diameter before drying (0.4 mm) / fiber diameter after drying (0.14 mm)). The fiber was dry for the confocal imaging.

[b] Larger sized CNC batch was used for the 6 and 9 wt.% fibers while the smaller sized CNC batch was used for the 12 wt.% fiber.
